# Harbingers of Plaque Instability: Dynamic ST-Elevation and Nonsustained Ventricular Tachycardia on Remote Monitoring

**DOI:** 10.1016/j.jscai.2025.102637

**Published:** 2025-03-25

**Authors:** Zachary Demertzis, Daniel Tim, James A. Goldstein

**Affiliations:** Department of Cardiovascular Medicine, William Beaumont University-Corewell Health, Royal Oak, Michigan

**Keywords:** coronary artery disease, nonsustained ventricular tachycardia, physiology, plaque, remote monitoring, ST-segment elevation myocardial infarction

## Abstract

Coronary atherosclerosis is a chronic, multifocal pathophysiologic process punctuated by acute inflammatory flares causing plaque destabilization. Plaque destabilization may smolder over a subacute temporal course, which may be clinically silent with spontaneous lesion healing resulting in multilayered plaques. We report a case who initially presented with acute coronary syndrome with culprit vessel revascularization and residual nonculprit disease who developed arrhythmogenic threat and dynamic ST elevations on remote monitoring that required emergent revascularization of the nonculprit vessel.

## Case presentation

**Figure 1:**
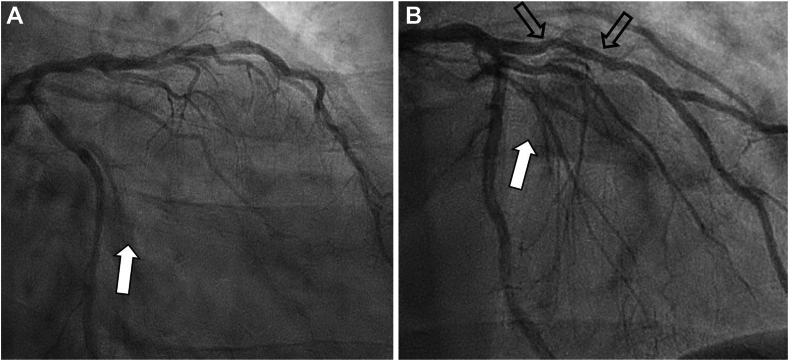
**Coronary angiography—June 2024.** Occluded obtuse marginal branch successfully stented (**A** and **B**, white arrows). Stable appearing left anterior descending artery stenosis of approximately 50% (**B**, open arrows).

**Figure 2:**
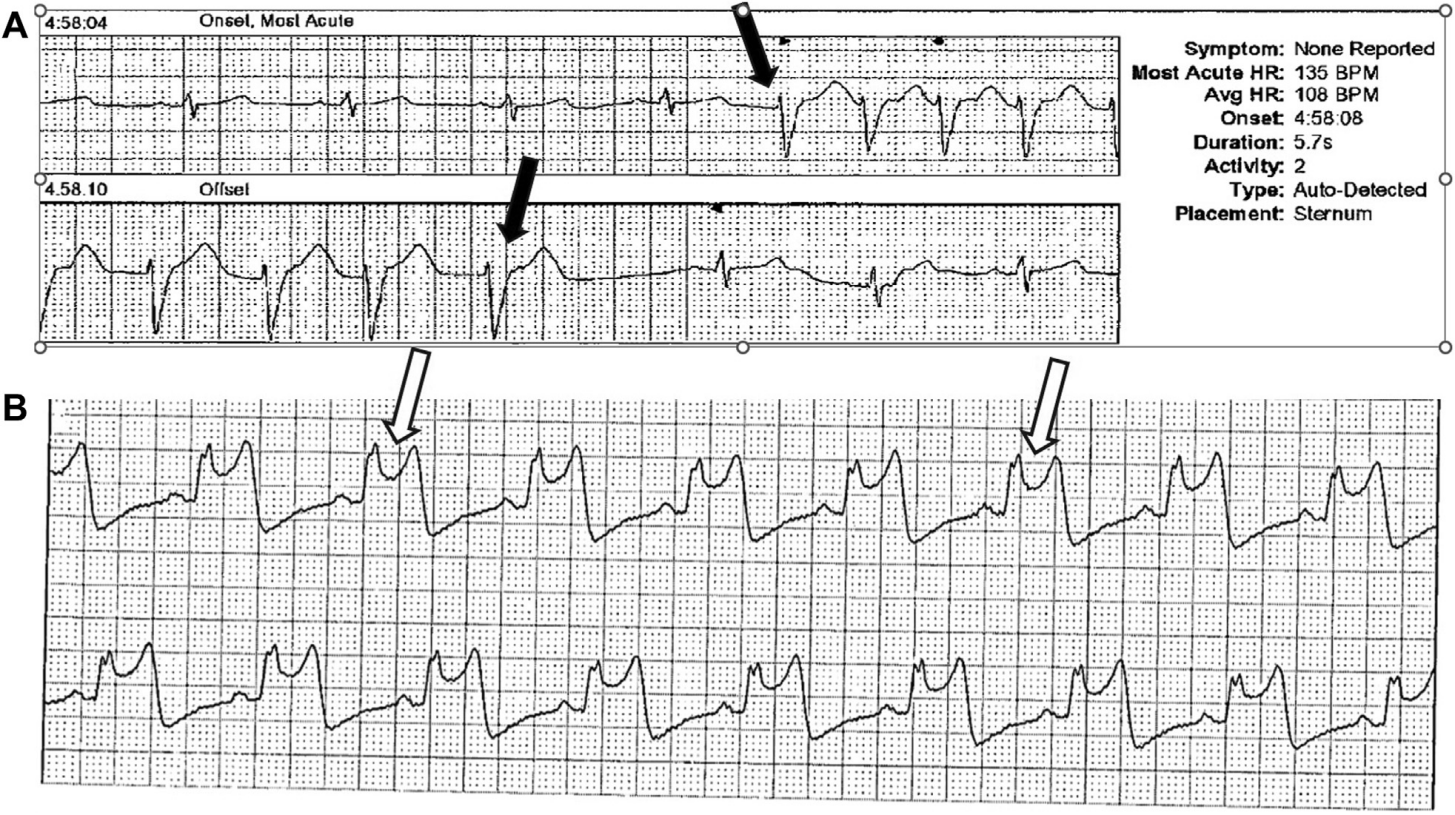
**Event monitor with (A) asymptomatic NSVT (black arrows) and (B) symptomatic ST elevations (open arrows)**. NSVT, nonsustained ventricular tachycardia.

**Figure 3:**
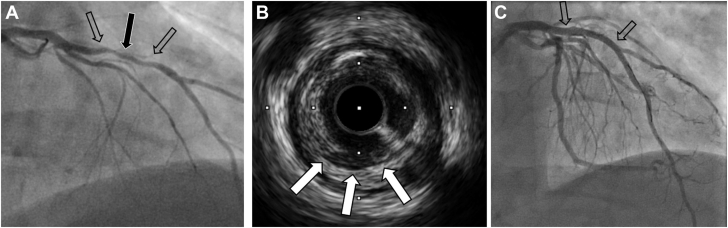
**Coronary angiography—November 2024.** (**A**) The obtuse marginal stent is patent; the LAD artery lesion progressed, both more stenotic (70%, open arrows) and with mid-lesion haziness (black arrow). (**B**) Intravascular ultrasound documenting mid-LAD plaque ulceration (white arrows). (**C**) Coronary angiography after successful stenting (open arrows). LAD, left anterior descending

A 50-year-old man presented in June 2024 with acute coronary syndrome (ACS); the culprit small occluded obtuse marginal (OM) branch was stented (Figure 1A, B, white arrows), whereas the left anterior descending (LAD) artery showed moderate 50% stenosis (Figure 1B, open arrows), and left ventricular systolic function was normal. Subsequently, palpitations led to an event monitor, which documented asymptomatic nonsustained ventricular tachycardia (NSVT) (Figure 2A). Despite β-blocker therapy, palpitations continued, and a 14-day Holter monitor documented dynamic ST elevations (Figure 2B), which, in retrospect, the patient correlated with episodic chest pain. Immediate triage to the emergency department showed normal electrocardiogram and negative high-sensitivity troponins. Angiography documented the OM stent patent (Figure 3A, open arrow); however, the mid-LAD lesion stenosis had progressed to approximately 70% and exhibited haziness (black arrow) consistent with plaque instability, confirmed by intravascular imaging documentation of plaque ulceration (Figure 3B, white arrows); stenting result was excellent (Figure 3C).

## Discussion

This case illustrates principles and patterns of plaque instability, which may be clinically abrupt or indolent and often manifests as a multifocal process attributable to inflammatory plaque destabilization.^1^ Coronary atherosclerosis is a chronic pathophysiological process punctuated by acute flares. The proximate cause of ACS is plaque destabilization with thrombus formation upon a disrupted, typically inflamed lipid-rich plaque. Intracoronary thrombus is a dynamic and variable process of clot formation, dissolution, and fragmentation, which may evolve over minutes or days culminating in abrupt occlusion, which may be sustained or undergo spontaneous thrombolysis, yielding a spectrum of clinical ACS manifestations based on the totality of occlusion. Although ACS is clinically abrupt, in many cases, it represents the clinically manifest end stage rather than the pathological initiation of plaque instability. Plaque destabilization may smolder over a subacute temporal course, which may be clinically silent with spontaneous lesion healing resulting in multilayered plaques.^2^, ^3^, ^4^ Plaque destabilization and the associated dynamic thrombus activity may precipitate sudden cardiac death.^2^^,^^3^

The present case illustrates such an arrhythmic threat. Initially presenting with ACS due to thrombotic circumflex culprit lesion, the LAD artery appeared angiographically stable. Yet, despite culprit vessel stenting, subsequent harbingers of intracoronary thrombus activity in the LAD were manifested as NSVT and episodic chest pain with dynamic ST elevations, consistent with intermittent embolization and occlusion. Repeat angiography documented that the LAD was more stenotic with evidence of destabilization shown by lesion haziness and confirmed by intravascular ultrasound delineated plaque ulceration, which is the substrate for such thrombus generation. It may be postulated that the original stable appearing LAD artery lesion may have been in the process of smoldering plaque destabilization undetected angiographically. This is consistent with the concept that ACS is typically due to plaque destabilization attributable to several mechanisms, including fibrous cap rupture or erosion, and intraplaque hemorrhage. Plaque disruption is often an acute exacerbation of an inflammatory response, a notion supported by pathologic prominence of inflammatory cell infiltrate in destabilized plaques and elevated systemic markers (eg, C-reactive protein). Factors postulated to promote plaque destabilization such as inflammation would tend to exert adverse effects throughout the atherosclerotic coronary tree. Given that coronary atherosclerosis is rarely focal, it therefore follows that plaque instability would not necessarily occur in isolation within a diffusely disease coronary bed. In ACS, inflammation is pan-coronary and plaque instability often multifocal, with clinical and pathological studies demonstrating that 50% of cases harbor a multiplicity of lesions either frankly disrupted or in the destabilization process.^5^

In aggregate, these observations support the concept that patients with ACS are “hot” and at greater risk for future nonculprit events. Delineating which non–flow-limiting lesions are vulnerable and at risk is key. Advances in intracoronary imaging now provide high-resolution characterization of plaque morphology and composition, detecting even subtle plaque disruptions and lipid-rich vulnerable plaques.^6^, ^7^, ^8^ Noninvasive screening with computed tomography angiography has potential to identify patients and plaques at risk.^1^ Implanted devices that detect dynamic ST elevations^9^ have interesting potential for real-time long-term monitoring. The implicit hope is that prospective identification and pacification of vulnerable lesions can improve clinical outcomes.^8^
